# Pharmacological Blockade of Group II Metabotropic Glutamate Receptors Reduces the Incidence of Brain Tumors Induced by Prenatal Exposure to N-ethyl-N-nitrosourea in Rats

**DOI:** 10.2174/1570159X23666241209090326

**Published:** 2024-12-10

**Authors:** Antonietta Arcella, Marika Alborghetti, Anna Traficante, Maria Antonietta Oliva, Sabrina Staffieri, Veronica Russo, Matteo Caridi, Giuseppe Battaglia

**Affiliations:** 1 IRCCS Neuromed, 86077 Pozzilli (IS), Italy;; 2 Department of Neurosciences, Mental Health and Sensory Organs (NESMOS), Sapienza University of Rome, 00185 Rome, Italy;; 3 Division of Hematology and Clinical Immunology, Department of Medicine, University of Perugia, 06125 Perugia, Italy;; 4 Department of Physiology and Pharmacology, Sapienza University of Rome, 00185 Rome, Italy

**Keywords:** Glioblastoma multiforme, glutamate, metabotropic glutamate receptors, N-ethyl-N-nitrosourea, rats, brain tumors

## Abstract

**Background:**

The study demonstrates that pharmacological blockade of type 3 metabotropic glutamate (mGlu3) receptors at the time of tumor induction significantly reduces the incidence of brain gliomas in rats. The overall survival of patients with high-grade brain gliomas is 14-20 months after current multimodal therapy, including surgery, radiotherapy, and adjuvant chemotherapy.

**Objective:**

To demonstrate in this experimental model that pharmacological blockade of group II metabotropic glutamate receptors reduces the incidence of brain tumors induced by prenatal exposure to N- ethyl-N-nitrosourea (ENU) in rats.

**Methods:**

Dams received a single injection of ENU (40 mg/kg, e.v.) at day 20 of pregnancy, combined with 5 daily injections of either saline or the mGlu2/3 receptor antagonist, LY341495 (10 mg/kg) (from day 15 to day 21 of pregnancy). Assessment of brain tumors in the offspring at 5 months of age showed the presence of mixed gliomas (astrocytomas/oligodendrogliomas) in 70% of the ENU + saline group of rats and only in 30% of the ENU + LY341495 group.

**Conclusion:**

Tumors in both groups of rats showed a moderate/high expression of the astrocyte marker, GFAP, and the oligodendrocyte marker, OLIG-2, and a low expression of the proliferation marker, Ki-67. However, tumors of the ENU + LY341495 group showed a reduced density of Iba-1^+^ cells, suggesting a lower extent of neuroinflammation in the tumor microenvironment. These findings strengthen the hypothesis that mGlu3 receptors are candidate drug targets for the treatment of malignant gliomas.

## 1 INTRODUCTION

Pharmacological blockade of type 3 metabotropic glutamate (mGlu3) receptors has been proposed as a novel therapeutic strategy for the treatment of brain gliomas [1-7]. The mGlu3 receptor belongs to the second group of mGlu receptor subtypes (together with the cognate receptor, mGlu2), is coupled to Gi/o proteins, and is found in neurons, astrocytes, oligodendrocytes, and microglia. Activation of mGlu3 receptors triggers multiple signal transduction pathways, such as inhibition of adenylyl cyclase activity and activation of the mitogen-activated protein kinase (MAPK) and phosphatidylinositol-3-kinase (PI3K) pathways [4, 8-10]. In addition, mGlu3 receptors are functionally coupled to mGlu5 receptors, and mGlu3 receptor activation boosts mGlu5 receptor signaling [11-14]. mGlu3 receptors are expressed by glioma cells and glioma stem cells (GSCs) [1, 14], and their activation sustains the undifferentiated state of GSCs by negatively modulating bone morphogenetic protein (BMP) receptor signaling, as assessed by SMAD phosphorylation and nuclear translocation [3]. In addition, mGlu3 receptor activation induces the expression of O6-methylguanine methyltransferase (MGMT) through the sequential activation of PI3K and nuclear factor-kB (NFkB) [4]. MGMT de-alkylates DNA, causing GSC resistance to temozolomide and other DNA alkylating agents [15]. By restraining this mechanism, mGlu3 receptor antagonists, LY341495 and LY2389575, enhanced GSC vulnerability to the cytotoxic action of temozolomide [4]. These findings were replicated using glioblastoma cell lines purified from surgical specimens, in which only the combination of temozolomide plus LY341495 caused cell toxicity [7]. *In vivo* data confirmed the synergism between LY341495 and temozolomide in reducing the growth of brain tumors generated by intracerebral infusion of GSCs in nude mice [4]. Synergism was also demonstrated in an elegant model in which temozolomide-resistant glioblastoma cell lines were applied to human cortical organotypic sections. The application of temozolomide plus LY341495 reduced tumor growth and caused substantial alterations in tumor cell morphology [7]. These findings encourage the combination of mGlu3 receptor antagonists in the treatment of brain gliomas. What is less clear is how these drugs affect glioma cell proliferation regardless of the presence of temozolomide or other DNA alkylating agents. Early studies showed that mGlu3 receptor blockade with LY341495 reduced the proliferation of human glioma cells in culture by limiting the activation of the MAP kinase and PI3K pathways [1, 14] and continuous s.c. delivery of LY341495 for 28 days reduced tumor growth after s.c. implantation or intracerebral infusion of U87MG human glioma cells in mice [1]. However, the antiproliferative effect of LY341495 in culture was not confirmed using glioma cells isolated from surgical specimens [7], and treatment with LY341495 alone did not significantly reduce the growth of brain tumors generated by intracerebral infusion of human GSCs in mice [4]. Here, it was decided to examine the effect of mGlu3 receptor blockade on gliomagenesis using the N-ethyl-N-nitrosourea (ENU) model in rats. ENU causes DNA ethylation and is a potent neurocarcinogen in rodents. Rats are more susceptible than mice [16, 17], and fetuses are 50-100 fold more susceptible than adult rats [18]. A single injection of appropriate doses of ENU (>20 mg/kg) to pregnant rats (12-22 days of pregnancy) induces the development of differentiated gliomas (astrocytomas, ologodendrogliomas, and mixed gliomas) in the offspring after a latency period of 3-4 months [19]. This model allows the study of drugs targeting early events in cellular transformation and neurocarcinogenesis. It is reported that the pharmacological blockade of mGlu3 receptors with LY341495 at the time of ENU injection in pregnant rats largely reduces the incidence of brain tumors in adult offspring.

## 2 MATERIALS AND METHODS

### 2.1 Drugs

ENU (Sigma-Aldrich, Milan, Italy) and (2S)-2- Amino-2-[(1S,2S)-2-carboxycycloprop-1-yl]-3-(xanth-9-yl) propanoic acid (LY341495) (Tocris, Bristol, United Kingdom) were dissolved in saline solution.

### 2.2 Animals’ Treatment

Adult Sprague-Dawley rats of both sexes (Charles River, Calco, Italy) were housed 3 per cage (one female and two males) under standard conditions with a 12:12 h light-dark cycle and food and water *ad libitum*. Day 1 of pregnancy was determined by observation of a vaginal plug. Two pregnant rats were used in the experiment. Dams received one single injection of ENU (40 mg/kg, e.v.) on day 20 of pregnancy. LY341495 (10 mg/kg) or saline was injected daily i.p. in ENU-treated dams from day 15 to day 21 of pregnancy. Pups were maintained with the mother until weaning (postnatal day 21), and then males and females were housed separately (4 per cage). Rats were killed at 5 months of age for the analysis of brain tumor. Two rats of the ENU/saline group and one of the ENU/LY341495 group died at 4-5 months of age and were not considered in the analysis. The study was conducted in line with international and national regulations with the 3Rs concept. All efforts were made to minimize animal suffering and to reduce the number of animals.

### 2.3 Histological and Immunohistochemistry on Rat Brain Tissue

Rats were transcardially perfused with 4% paraformaldehyde (PFA) in 0.1 M phosphate buffer, pH 8. Dissected brains were post-fixed overnight in 4% PFA at 4°C and then cryopreserved by immersion in 30% sucrose in PBS. Brains were paraffin-embedded, and 0.5 μm thick serial sections (1 section every 400 μm) were cut. Sections were stained with Mayer’s hematoxylin and eosin (H&E) (both from Diapath, Bergamo, Italy) and subjected to analysis for the quantification of tumor volume. Volumetric analysis was performed by software measuring the tumor area in each section and calculating the volume of the tumor according to Cavalieri’s method, using the following formula:

V = ∑(A)i × TS × n,

where (A)I is the area of the tumor in level i, TS is the section thickness, and n is the number of sections between the two levels [20]. Immunohistochemical analysis was carried out on 4 µm thick sections using an automatic immunostaining Benchmark ultra-XT (Ventana). An Ultra View DAB Detection Kit (Roche Diagnostic, Mannheim Germany) was used with mouse anti-GFAP monoclonal antibodies (prediluted; Roche Diagnostic, Mannheim Germany), mouse anti-SYP monoclonal antibodies (1:100, Thermo Fisher, Italy), mouse anti-Ki-67 monoclonal antibodies (Recombinant Monoclonal Antibody SP6; 1:100, Thermo Fisher, Italy), mouse anti-IBA1 monoclonal antibodies (1:200, Fujifilm Wako, chemical U.S.A. corporator) and mouse anti-OLIG2 monoclonal antibodies (1:200, Merck KGaA, Darmstadt, Germany). Slides were analyzed by an optical microscope at 20X and 40X magnification.

## 3 RESULTS

**Fig. (1):**
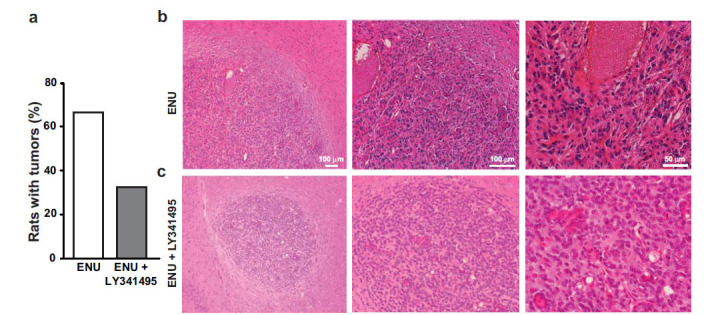
The offspring of pregnant dams injected with ENU and treated with saline or LY341495. Percent of rats developing brain tumors (**a**). Representative images of H&E staining of tumor sections in a rat injected with ENU and treated with saline (**b**) and a rat injected with ENU and treated with LY341495 (**c**).

**Fig. (2):**
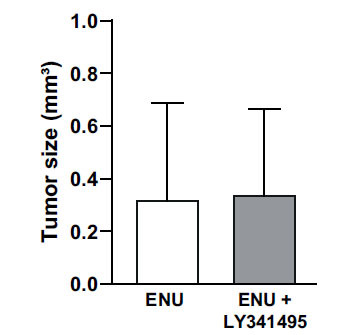
Volumetric analysis of the tumor size in rats injected with ENU and treated with saline or LY341495.

**Fig. (3):**
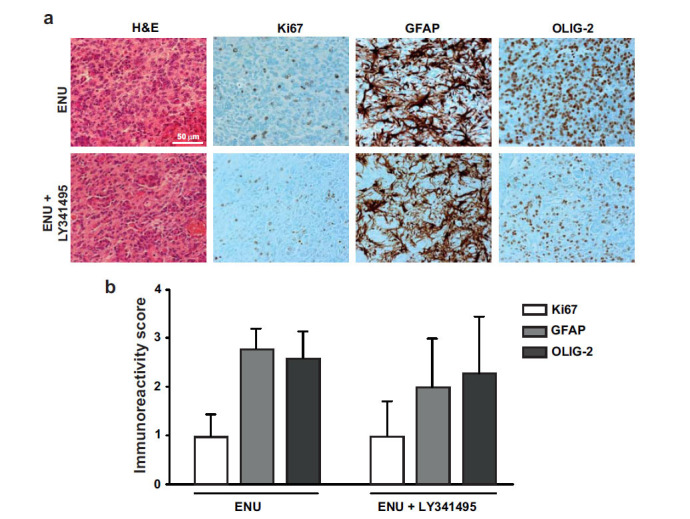
Representative images of H&E staining and immunohistochemistry for Ki67, GFAP, and OLIG-2 in a rat injected with ENU and treated with saline and a rat injected with ENU and treated with LY341495 (**a**). Immunoreactivity score of GFAP, OLIG2, and Ki67 in rats injected with ENU and treated with saline or LY341495 (**b**).

**Fig. (4):**
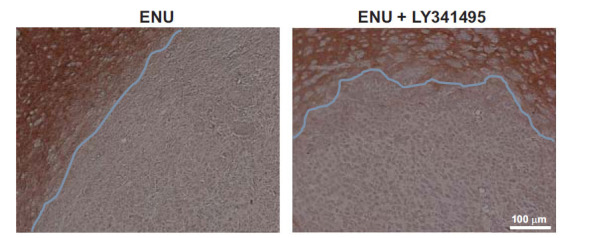
Representative immunohistochemistry for synaptophysin in a rat injected with ENU and treated with saline and a rat injected with ENU and treated with LY341495. The tissue sections underwent immunohistochemistry for GFAP and SYP.

**Fig. (5):**
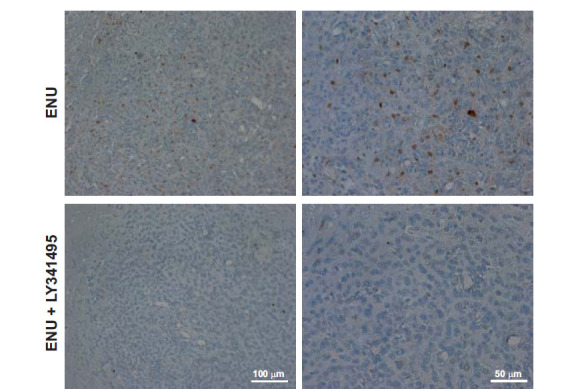
Representative images of immunohistochemistry for IBA1 in a rat injected with ENU and treated with saline and a rat injected with ENU and treated with LY341495. Note that immunoreactivity for IBA1 (brown) is present only in rats injected with ENU.

Analysis of brain tumors was carried out in the adult offspring of dams receiving a single injection of ENU (40 mg/kg) at day 20 of pregnancy and 5 injections of LY341495 (10 mg/kg) or saline from day 15 to day 21 of pregnancy. Ten rats per group were killed at 5 months of age, and, at that time, none of the animals showed motor impairment. H&E staining showed large brain tumors in 70% of rats of the ENU + saline group (Fig. **1a**). Tumors showed pleomorphic cell morphology. Cell nuclei appeared voluminous, vesicular, and condensed, with a compacted nuclear domain and relevant nucleoli. Cells were arranged in mutual contact in a disordered fashion and were devoid of cohesion. There was no evidence of necrosis, and the number of mitoses was very low in the tumor mass of both groups (Figs. **1b** and **1c**). Interestingly, ENU + saline group morphology showed mild nuclear atypia, condensed cytoplasm, more elongated or irregular shape, and prominent vascularization, which is typical of higher malignancy glioma forms. Only 30% of rats of the ENU + LY341495 group developed brain tumors at 6 months of age (Figs. **1a** and **1c**). A densitometric study of the extent of tumors was performed using a stereomicroscope. By evaluating the extension of the tumor area in mm^3^, most of the tumors were unilateral, showed a preferential location in the dorsal striatum, and tumor volume ranged from 0.03 to 1.02 mm^3^. Extension of the glial lesion was similar in the two groups, although fewer gliomas developed in the progeny of LY341495-treated rats (Fig. **2**). Intensity of immunoreactivity for the astrocyte marker, GFAP, the oligodendrocyte marker, OLIG-2, and the proliferation marker, Ki-67, was graded as 0 (absent), 1 (low), 2 (moderate), or 3 (high). GFAP and OLIG-2 immunostaining were scored as 2 or 3 in all tumors of the ENU + saline group, whereas Ki-67 staining was low. GFAP, OLIG-2, and Ki-67 immunoreactivity was also comparable in the two groups of rats, although it was more heterogeneous in the ENU + LY341495 group (Fig. **3a** and **3b**). The proliferative index evaluated with Ki-67 was about 7% in tumors of both groups. Immunoreactivity for synaptophysin (SYP), a neuronal marker of presynaptic terminals, appeared only at the border of the tumor (Fig. **4**). Thus, ENU-induced tumors had the appearance of differentiated mixed gliomas with a low proliferation rate. The expression of the ionized calcium-binding adapter molecule 1 (IBA1), which is up-regulated in activated macrophages and microglia, was also evaluated [21]. We found lower IBA1 immunoreactivity in tumors of the ENU + LY341495 group (Fig. **5**), suggesting a reduced inflammatory response in tumors of this group.

## 4 DISCUSSION

Our findings demonstrate that early pharmacological blockade of mGlu3 receptors with the orthosteric antagonist, LY341495, largely reduced the incidence of brain tumors induced by prenatal exposure to ENU in rats. LY341495 displays high affinity for both mGlu2 and mGlu3 receptors, but only mGlu3 receptors are expressed in astrocytes and glioma cells [10]. Remarkably, mGlu3 receptors were blocked at the time of ENU injection, suggesting that endogenous activation of mGlu3 receptors was necessary for the induction of neurocarcinogenesis. One possible explanation is that endogenous activation of mGlu3 receptors maintains the undifferentiated state of GSCs, which become committed to slowly proliferate, migrate, and give rise to a brain glioma at a site near the periventricular region, *i.e*., the dorsal striatum. Accordingly, *in vitro* data have shown that mGlu3 receptor activation restrains GSC differentiation by negatively modulating the action of BMPs [3]. Consistent with the literature, a single injection of 40 mg/kg of ENU in pregnant dams pre-treated with saline induced the development of brain tumors in 70% of the adult offspring (see Introduction and References therein). These tumors showed proliferation and no signs of necrosis but vascolarization, suggesting a high grade of malignancy. Early blockade of mGlu3 receptors with LY341495 did not cause visible changes in the phenotype of brain tumors in the few rats (3 out of 10) that responded to prenatal exposure to ENU. However, tumors induced by ENU in the presence of mGlu3 receptor blockade showed a reduced number of activated macrophages/microglia, as indicated by a lower Iba-1 immunoreactivity. Macrophages and microglia are the major infiltrating immune cells of the tumor microenvironment and interact with glioma cells, supporting glioma cell proliferation, migration, and survival [22]. Activated macrophages/microglia release pro-inflammatory cytokines, *e.g*., IL-1, IL-6, and IL-8, which contribute the progression of malignant gliomas [22, 23]. Thus, one can predict that the few tumors originating in response to ENU when mGlu3 receptors are inhibited have a better prognosis. Our data integrate previous studies and fully support the hypothesis that mGlu3 receptors are involved in the pathophysiology of brain gliomas. Computational analysis led to the identification of a putative new subtype of malignant glioma characterized by a high expression of mGlu3 and mGlu5 receptors [24]. Two independent studies have demonstrated that mGlu3 receptor expression in surgical samples of glioblastoma was directly related to malignancy and tumor recurrence and inversely related to response to temozolomide and patients’ survival. Ciceroni *et al*. [4] measured the transcript of mGlu3 receptors in glioblastoma samples obtained from 87 patients undergoing surgery, followed by adjuvant therapy with temozolomide. Overall survival was longer in patients with a tumor expressing low levels of mGlu3 receptor mRNA, and methylation of the MGMT gene promoter prolonged survival only in those patients showing low levels of mGlu3 mRNA in the tumor. More recently, Maier *et al*. [7] measured mGlu3 receptor expression in glioblastoma samples removed from 12 patients undergoing a combined radio- and chemotherapy protocol. Receptor expression was greater in recurrent tumor samples than in *de novo* tumor samples, and overall survival was higher in patients with low mGlu3 receptor-expressing tumors. We believe that it is the right time to develop selective mGlu3 receptor antagonists or negative allosteric modulators for the treatment of malignant gliomas. These drugs, combined with the current treatment strategies, are predicted to restrain tumor growth and reinforce the therapeutic efficacy of DNA-alkylating agents. In addition, measurements of mGlu3 receptor expression in tumor specimens may have prognostic values in patients with malignant gliomas.

## CONCLUSION

The study found that early pharmacological blockade of mGlu3 receptors with the orthosteric antagonist, LY341495, reduced the incidence of brain tumors in rats induced by prenatal exposure to ENU. This suggests that endogenous activation of mGlu3 receptors is necessary for neurocarcinogenesis. This study also served to identify a new subtype of malignant glioma characterized by high expression of mGlu3 and mGlu5 receptors that have been independently reported [24]. The researchers believe it is time to develop selective mGlu3 receptor antagonists or negative allosteric modulators for the treatment of malignant gliomas.

## Data Availability

Not applicable.

## LIST OF ABBREVIATIONS

ENU: N-ethyl-N-nitrosourea
GSCs: Glioma Stem Cells
MAPK: Mitogen-activated Protein Kinase
mGlu3: Type 3 Metabotropic Glutamate
MGMT: O^6^-methylguanine-DNA Methyltransferase
NFkB: Nuclear Factor-kB
PI3K: Phosphatidylinositol-3-Kinase
